# Can Deliberative Participatory Fora Cure Representation Gaps in France and Germany?

**DOI:** 10.1007/s11615-022-00452-0

**Published:** 2023-01-30

**Authors:** Agnes Blome, Miriam Hartlapp

**Affiliations:** Otto Suhr Institute of Political Science, Freie Universität Berlin, Ihnestr. 22, 14195 Berlin, Germany

**Keywords:** Descriptive representation, Social groups, Political inequality, Democracy, Citizen assemblies, Deskriptive Repräsentation, Soziale Gruppen, Politische Ungleichheit, Demokratie, Bürgerräte

## Abstract

The French and the German national parliaments are dominated by highly educated, older, and mostly male politicians. There are growing calls for a more balanced political representation of different social groups. This paper seeks to inform this debate by conceptualizing and measuring representation gaps for women, people of immigrant origin, the working class, and younger age groups in France and Germany and by assessing the potential of deliberative participatory fora to ameliorate underrepresentation. Based on theories of deliberative and participatory democracy, it suggests three criteria these fora must fulfill to potentially balance underrepresentation (descriptive representation in composition, deliberative quality, and coupling to politics) and explores them empirically in four recent cases of deliberative participatory fora: the *Grand Débat National* and the *Convention Citoyenne pour le Climat* in France and the *Bürgerrat Deutschlands Rolle in der Welt *and the *Bürgerrat Klima* in Germany. We show that significant representation gaps exist for all groups studied. They have been narrowing for women and people of immigrant origin and remain most pronounced for class. Regarding institutional features, our cases fare relatively well in terms of balanced composition and deliberative quality, but the potential to balance representation gaps is seriously limited by a lack of coupling to the political system.

## Abstract (de)

In der französischen Assemblée Nationale wie im deutschen Bundestag sind mehrheitlich höher gebildete, ältere und männliche Politiker*innen vertreten. Seit einiger Zeit wird eine ausgewogenere politische Repräsentation verschiedener sozialer Gruppen gefordert. Der Beitrag dieses Artikels zielt auf die Konzeptualisierung und Messung von Repräsentationslücken für Frauen, Menschen mit Migrationshintergrund, Arbeiter:innen und jüngere Menschen in Deutschland und Frankreich und versucht, das Potenzial deliberativ-partizipativer Verfahren zur Verringerung der Repräsentationslücken zu erfassen. Basierend auf Theorien deliberativer und partizipativer Demokratie werden drei institutionelle Kriterien vorgestellt, die diese Verfahren erfüllen müssen, um existierende Repräsentationslücken auszugleichen (deskriptive Repräsentation in der Zusammensetzung, deliberative Qualität und Verbindung zur Politik). Vier aktuelle deliberativ-partizipative Verfahren werden anschließend anhand der Kriterien untersucht: die *Grand Débat National* und die *Convention Citoyenne pour le Climat* in Frankreich sowie der *Bürgerrat Deutschlands Rolle in der Welt *und der *Bürgerrat Klima* in Deutschland. Wir zeigen, dass für alle analysierten Gruppen relevante Repräsentationslücken bestehen, die für Frauen und Menschen mit Migrationshintergrund im Untersuchungszeitraum kleiner werden, während sie für Arbeiter:innen am größten sind. Die institutionelle Gestaltung der untersuchten deliberativ-partizipativen Verfahren kann die repräsentative Zusammensetzung und Qualität der Deliberation fördern, das Potenzial zum Ausgleich von Repräsentationslücken wird aber durch die schlechte Verbindung zum politischen Entscheidungsprozess stark begrenzt.

## Introduction

Inequality of representation has been identified as a major challenge to liberal democracies. The development both of political parties and of national parliaments toward a “diploma democracy” (Bovens and Wille 2017) characterized by highly educated, older white men has raised fears that the interests of other groups in society—e.g., women; people of immigrant origin; and people who are disabled, young, or have a low level of education—do not play a role in politics.

Existing research has contributed to understanding the drivers and consequences of unequal representation. Arguably, from both perspectives the gender representation gap has been studied most widely (e.g., Murray 2016; Elsässer and Schäfer 2018), but other social groups have come into view, e.g., ethnic minorities (Bloemraad 2013; Geese 2022) and younger age groups (Stockemer and Sundström 2018; Freire et al. 2022). Analyses tend to focus on single groups, and methodologically, a national perspective prevails. This limits insights on broader trends of narrowing or widening representation gaps as well as potential trade-offs between groups. What is more, so far scholarly attention has focused mostly on capturing and explaining the unequal political representation and its implications for democracy. The logical next step is to turn to the institutional responses to these challenges, as they will be crucial in determining the impact that current developments will have on the working of Western democracies in the future. Where institutional responses to face representation gaps are studied, this mainly concerns parity legislation and quotas (Krook 2010) and, thus, changes addressing the electoral process. We build on these insights and ask for institutional innovations that aim at balancing representation gaps, but we look at extraparliamentary fora. Much hope is placed on deliberative participatory fora, i.e., fora “in which citizens participate in making collective decisions through deliberation” (Elstub 2018, p. 187). What features are necessary to ensure that these new types of citizen involvement have the potential to balance existing inequalities in representation? How are they implemented in practice?

We argue that to address these questions, it is necessary to first identify the representation gaps for multiple groups. We focus on legislators as central institutions of democracies and decisive actors for policy-making. Second, we assess institutional features of deliberative participatory fora to understand their potential to balance existing deficits. We compare representation gaps and institutional responses between France and Germany. Our choice of these two countries was motivated by the similarities in group representation deficiencies and respective efforts to address these via deliberative participatory fora. At the same time, the two countries are characterized by differences in their electoral systems and cultural framings of representation that matter for the political representation of social groups. This enables us to generalize conclusions to countries with varying politico-institutional features.

In the following section we develop our theoretical approach to political representation and deliberative democracy. We describe how the measurement of the representation gaps, the selection of groups and countries should be assessed, as well as three institutional criteria that have to be fulfilled for deliberative participatory fora to tackle underrepresentation by opening new channels of participation. Next, we illustrate these gaps for women, ethnic minorities, the working class, and younger age groups in France and Germany, based on primary and secondary sources. Finally, we empirically explore the deliberative participatory fora’s potential in four recent cases: the *Grand Débat National* and the *Convention Citoyenne pour le Climat* in France and the *Bürgerrat Deutschlands Rolle in der Welt* and the *Bürgerrat Klima* in Germany. We conclude with a critical assessment of the relationship between parliamentary and extraparliamentary forms of representation.

## Political Representation and Deliberative Democracy—Theoretical Framework and Empirical Approach

### Group-Specific Political (Under)Representation

The study of political representation has received great attention in theoretical and empirical research on democracy. The main question is whether and how citizens’ preferences are represented in policy-making by actors who speak and act on behalf of citizens. For a long time, the view of political parties channeling alternative policy visions based on their constituents’ interests prevailed (Rohrschneider and Thomassen 2020). Scholars have also explored more direct links between citizens’ preferences and elected political representatives. These studies typically focus on sociodemographic characteristics and actions on part of the representatives. One of the most cited conceptualizations identifies formalistic, descriptive, substantive, and symbolic forms of representation (Pitkin 1967). While formalistic representation refers to the institutional arrangements of how representatives obtain office and how constituents may hold them accountable (e.g., by voting them out of office), symbolic representation concerns the meaning a representative has for the constituents. Descriptive representation is the extent to which representatives are similar to those represented in terms of sociodemographic characteristics, interests, and experiences. Lastly, substantive representation refers to the actions of representatives on behalf of the represented, i.e., the extent to which representatives advance the constituents’ preferences in policy-making. Pitkin’s work has been highly influential, especially for debates on whether marginalized groups in society need representatives from their groups (Dovi 2018).

The concept of political representation has been further developed to account for broader and multiple forms of the relationship between the representatives and the represented, pointing in particular to the context dependency of interests and the way representatives create and frame claims put forward by the represented (importantly, Mansbridge 2003; Saward 2010). Recently, these more constructivist conceptions of representation have found their way into quantitative social research on the quality of representation (Wolkenstein and Wratil 2021), yet they fall short of providing a picture of the diversity of parliaments.

The burgeoning literature on inequalities in representation, however, still makes the case for analyzing to what extent legislators mirror groups in society—even without showing that their inclusion *in fact* makes a difference to policy outcomes. In spite of criticism of the term “descriptive representation” as being too passive, the concept uniquely enables exploration of which groups in society are marginalized in political decision-making. This is because of the premise that legislators are not robots who act on what their constituents expect from them. Rather, their social background likely makes a difference. As Phillips put it, “the knowledges they draw on from their social experiences become relevant to their political decisions” (Phillips 2020, p. 179). For example, legislators from upper classes vote differently than their working-class colleagues do even when they belong to the same left-wing party (O’Grady 2019) or demonstrate that they understand working-class interests (Carnes 2013). In other words, the presence of marginalized groups in parliaments more likely leads to a transformation of policies in favor of these groups (e.g., Wängnerud 2009; Elsässer and Schäfer 2018).

Research shows that when marginalized groups feel that established collective actors such as political parties no longer pursue their interests, they worry much more about descriptive representation of their group (Urbinati 2012). Also, groups that feel that their external efficacy is low might even refrain from political participation. Lower political participation of social groups is, however, associated with a lower likelihood that their interests matter in decision-making processes because legislators arguably pursue those policy outcomes that will reward them with votes (Elsässer and Schäfer 2022). In addition, descriptive representation matters as representatives act as role models in politics and society (Bühlmann and Schädel 2012).

So far, research on the causes and consequences of underrepresentation has largely focused on women and increasingly on people of immigrant origin. Researching a plurality of groups is conceptually demanding due to difficulties of defining in and out interests (e.g., Kroeber 2018) and empirically demanding due to lack of comparable data when it comes to, e.g., sexual orientation or religious beliefs in society and among representatives. Theoretically, the choice of which underrepresented groups are researched is justified with a focus on a) historically excluded and oppressed groups (prominently, Phillips 1995, pp. 174–175; Mansbridge 1999) and b) a proven lack of responsiveness to group interests, e.g., of the working class (Elsässer and Schäfer 2022) and younger age groups (Stockemer and Sundström 2018; see also Phillips 2020).^1^

In summary, it is broadly accepted that gaps in descriptive representation may pose a threat to democracy and that reaching more socially and demographically balanced parliaments is desirable (Mansbridge 1999). As explained by Bloemraad and Schönwälder for the case of people of immigrant origin (2013, p. 565), “Even if immigrants’ interests may be represented by politicians who are not of immigrant origin, the lack of diversity in Europe’s legislatures sends a message of exclusion and signals a democratic deficit within domestic politics.”

### How Deliberative Democracy May Compensate for Descriptive Underrepresentation

Given these intricacies of representative democracy, proponents of deliberative democracy suggest that innovative fora for policy deliberation may supplement the institutions of representative democracy. The concept of deliberative democracy connotes debates on political issues among equal and mutually respectful people who then decide on policies based on the discussions (Habermas 1997; Bächtiger et al. 2018). It thus involves talking and making choices based on these discussions. Deliberative democracy speaks to both citizens and political representatives. Deliberation helps “the citizens to understand better the issues, their own interests, and the interests and perceptions of others; forge agreement where possible; and, in the instances in which agreement is not possible, both structure and clarify the questions behind the conflict” (Bächtiger et al. 2018, p. 2). Deliberative democracy’s goal as well as its requirement is to give reasons and justify policy decisions. It is, however, unclear to what extent those who deliberate (and make choices) represent—stand for, speak for, act for—those who do not deliberate (Brown 2018). Scholarship that connects deliberative democracy and representation theories has thus warned against socially exclusive deliberative fora (Williams 1998). Rather, more diverse social perspectives should be represented in any deliberation, ideally by group members themselves. By doing so, some argue that deliberative participatory fora can function as “a cure for the current disenchantment with representative democracy” (Trüdinger and Bächtiger 2022, p. 1), and they stress the potential of innovation outside the political arena to “counteract failures of representative democratic institutions” (Kuyper and Wolkenstein 2019, p. 656). Scholarship highlights the link between the installment of such fora and the governments’ interest in healing electoral democracy and in demonstrating responsiveness to the demands of people (Macq and Jacquet 2023), while there are also more skeptical arguments (e.g., Lafont 2017; Font et al. 2018; different contributions in Bächtiger et al. 2018)—empirically, the jury is still out. Using the words of Vermeule (2012, p. 338), “the next task is to bring these ideas down to the level of institutional design.”

### Criteria to Assess the Potential of Deliberative Participatory Fora to Balance Representation Gaps

How should deliberative participatory fora be designed in order to balance representation gaps? Criteria lists have been established on the basis of best practice and expert input (Chwalisz 2020; Curato et al. 2021). We draw on these lists, focusing on criteria that directly inform our research questions on the potential to balance existing inequalities in representation and their implementation.^2^ Further, the selection of our three criteria follows the theoretical case for political representation and deliberative democracy (see above). Consequently, the first criterion is grounded in reasoning on descriptive representation and calls for composition of these fora to mirror society in terms of gender, class, ethnic minorities, and age. The second criterion builds on the idea that deliberative democracy of good quality must render underrepresented perspectives visible, integrate expertise, allow for learning, and come to widely accepted conclusions. The third criterion takes issue with a central premise of this article: We are interested in institutional innovations complementing existing democratic systems—thus, we have to assess their place in these systems. Fora must be coupled to the existing political institutions by a clear mandate and have follow-up procedures that can foster responsiveness of decision-taking in the political system.

#### How should the deliberative participatory forum be composed?

With acceptance of the theoretical case for descriptive representation, the representative composition of deliberative participatory fora becomes a necessary condition to balance representation gaps in parliaments. Historically, sortition (civic lottery) ensured that every member of a community had an equal chance of being selected, e.g., officials in ancient Athens or the Doge of Venice. To overcome a purely random sample and to ensure that the demographic composition of the sample matches that of the population, today sortition uses stratified sampling. Typically, strata of gender, age, migration, educational attainment, or territorial distribution are considered. Such selection processes are still biased by drawing in the more interested and available individuals in society, but since they refer to existing group-specific inequalities in political representation (see 2.1), they come closer to a mirror image of society. Balanced participation can be further enhanced through coverage of expenses, compensation for income loss as well as for child and elder care (Mansbridge 1999, p. 653), or through oversampling of certain demographic characteristics (e.g., Chwalisz 2020, p. 118). Size of the forum is also a concern, as larger settings are typically better when representing different group interests and bringing in multiple experiences (Curato et al. 2021).

#### What are favorable conditions for a high-quality deliberative process in the forum?

Habermas’s (1997) seminal theory on “epistemic democracy” claims that more inclusive deliberative processes are conjectured to lead to better decisions. The quality of deliberation is considered to be greater the more “informed, impartial, mutually respectful, and open to counter-arguments participants are” (Lafont 2017, p. 86). From this perspective, diversity of participants is not sufficient, but institutions must ensure that the diverse views are present in deliberations and are heard equally. Institutional features can support deliberation to reach these qualities: Participants should be provided with broad and diverse information and expertise (ideally, on demand to avoid agendas being simply taken over). It is important for the information to be accessible and to empower those representatives who might have entered the deliberative participatory forum with less information (Curato et al. 2021). Skilled facilitation in the form of professional and content-neutral moderation is also important to ensure that all interests are heard rather than reproducing existing imbalances in a group (Landwehr 2014). Here, a mix of formats (broad plenary discussion and small groups with greater privacy) is also beneficial as they are more likely to give every participant an opportunity to engage and exchange (Chwalisz 2020, p. 118). Furthermore, plenty of time is needed to ensure participation and iterative exchange over weeks or even months (Gherghina et al. 2021). Theoretically, the nature of deliberation as the result of an open exchange at eye level makes it difficult to deduce expectations about a generally valid “ideal” duration. Yet it is argued that when the process is stretched out too long, problems related to attrition or group socialization negatively affecting the equality of contributions might emerge (Curato et al. 2021). Therefore, we propose that the individual sessions should follow within weeks rather than within months. As for all complex processes, evaluation is important to ensure good practices throughout the process.

#### What ensures coupling of the deliberative participatory forum and politics?

Deliberative participatory institutions differ in the tasks they have. Some hold a clear mandate to formulate policy recommendations, whereas others are more loosely connected to the political system, e.g., when their function is to bring citizens’ latent opinions to the fore. We argue that the idea of balancing the unequal representation in parliaments requires deliberative participatory fora recommendations to hold a certain degree of bindingness to the political system. Recently, van Dijk and Lefevere (2022, p. 1) have shown that disregarding citizens’ recommendations emerging from these fora can “lead to more dissatisfaction than not asking for [their] advice” (see also Goldberg and Bächtiger 2022). Institutional features can help render this coupling more likely (Setälä 2017, pp. 853–857). On the demand side, a clear and delimited task as well as a mandate by political officials, e.g., a parliamentary committee, a ministry, or a president, is beneficial. In addition, the more different issues that participants must grapple with, the more likely an early saturation point that might make participants leave and might eventually lead to lower deliberative quality (Smith and Setälä 2018). Ultimately, a two-directional or even multidirectional coupling is warranted where a forum is linked to various relevant actors and institutions to interact and to exchange. Citizens would be “given the opportunity to question members and hold them to account” (Hendriks 2016, p. 56). On the output side, the citizens’ report or recommendations should have a clear purpose and address (e.g., parliament or government), and it should be explicit in specifying what steps will follow (e.g., a hearing in parliament, referral to other bodies) (Goodin and Dryzek 2006; Font et al. 2018). Otherwise, the fora’s recommendations might run the risk of going astray.

Analytically these three criteria are distinct. Yet we can consider balanced composition as a basic institutional feature that deliberative participatory fora must fulfill to have the potential to balance existing representation gaps. But even when this criterion is fulfilled, it is no cure for representation gaps where the quality of deliberation is low or where output can be easily ignored by established political institutions.

## Approaching Political Representation and Deliberative Participatory Democracy Empirically

Empirically we focused on France and Germany as two large European countries experiencing substantial political inequalities. Representation gaps as phenomena of interest have been identified in both countries and will be specified empirically in this paper. In both countries, we observe new dynamics of collective mobilization (e.g., Yellow Vests, Patriotic Europeans Against the Islamization of the Occident [PEGIDA]) that evoke calls for a more balanced political representation of different social groups as well as substantively interesting institutional responses to these calls (Grossman 2019; Trüdinger and Bächtiger 2022). In recent years, nationwide deliberative participatory fora have been established in both countries to debate issues of public administration, economy and fiscal policy, and democratic quality in general, and the issue of climate change in particular. Besides the similarities, France and Germany were also chosen because their electoral systems and cultural framings of representation work in ways that render persistent inequalities in political representation possible, yet they differ in important aspects. This case selection increases the possibilities to generalize findings to countries with similar politico-institutional features.

How the design of electoral institutions may shape the chances of candidates from different social groups has been shown in numerous studies (for an overview, see Paxton et al. 2010). In France, the 577 members of the Assemblée Nationale are elected in a two-round majoritarian system. The lack of proportionality is a central challenge for balanced representation, along with the practice of accumulating mandates (*cumul des mandats*). In 2000, after long political struggles, France introduced a legislative gender quota, the so-called parity law. Germany’s mixed-member electoral system combines elements of proportional and plurality systems. Typically, candidates from minority groups have higher chances to receive a promising list position than to run in a “winnable” district. Instead of legislative quotas, many parties apply party internal—formal and informal—quotas to their party list composition. These apply mainly to gender, age, and regions (Reiser 2014).

The French state is crafted around the “universal” or “republican” notion. This means that the state “refuses to recognize citizens by category, including sex and race, insisting on equality de jure even in the face of de facto inequalities” (Murray 2016, p. 587). Consequently, the Assemblée Nationale represents the nation in its totality, rather than its individual members. Data collection on race and immigrant origin is banned from the French census (Murray 2016, p. 587). These thoughts contrast with the idea of descriptive representation at large. In Germany, the individual member of parliament (MP) is committed to pursuing the goals of his or her party (as well as being committed to his or her conscience). In that sense, party affiliation may be more important for representatives than group belonging is. However, when a group has a higher descriptive representation, it may also be able to influence the definition of party goals (Kroeber 2021).

### Measurement of Representation Gaps

In a first step, we assessed the descriptive representation of different social groups that are known to suffer from underrepresentation more generally: women, ethnic minorities, working-class people, and younger citizens. A “representation gap” occurs when the share of legislators from a social group is lower than the group’s share in the general population. We followed Bloemraad (2013, pp. 657–658) and operationalized representation gaps with a representation index (RI) by “dividing the percentage of minority representatives in a particular elected body by the percentage of people from that same minority group among the general population. A figure of 0 indicates an absolute lack of representation while 1 indicates perfect ‘mirror’ representation: there is parity in the minority group’s proportion in the population and their proportion in the elected body. Numbers below 1 indicate underrepresentation; those above 1 signal more representation in office than we would expect based on demographic data alone.” The lower the RI of a social group, the more that compensation via other political channels becomes necessary—in our case, the balanced composition of deliberative participatory fora.

In the following, we define and conceptualize the groups affected by underrepresentation. Acknowledging that gender is a socially constructed category, we define women as those persons who are assigned female sex at birth, or as persons who define themselves as women. However, official population statistics may not always account for the latter while the legislators’ gender identity may be adapted. For example, in the current Bundestag two German legislators identify (and count) as women while they are assigned male sex at birth in the official population statistics.

Research on the representation of ethnic minorities typically uses one of three distinct concepts and corresponding measures: First, foreign population—defined as individuals with a non-national citizenship living in a country—is used as a proxy in administrative statistics. Yet when it comes to elections, in many countries foreigners do not have active or passive voting rights. What is more, a passport—at least when we think of European Union (EU) citizens living in another EU member state—does not capture shared discriminatory experiences and thus cannot bring into presence the information, perception, and insights valuable for democracy (Phillips 1995). Therefore, the validity of this measure is limited. Second, the concept of immigrant-origin population is used. It is defined as all individuals who themselves and whose parents (at least one) did not acquire national citizenship by birth (e.g., in the German microcensus). Yet as a consequence, indigenous and linguistic minorities, such as in the French overseas departments (in the Caribbean, Indian Ocean, and South Pacific Ocean) are not captured. These minorities are, however, theoretically relevant for descriptive representation as a mirror of society. A third concept, visible minority, arguably allows for better consideration of racial discrimination (Jenichen 2020). Visible minorities are typically assessed on the basis of a) name (surname, first name), b) photograph, and c) place of birth (and place of birth of ancestors) (Keslassy 2017, p. 21). The drawback is that the notion of visible minority is typically not used in representative surveys or administrative data. In addition, comparison is limited by the context specificity of the measure. To measure representation gaps for ethnic minorities, we therefore relied on the concept of immigrant-origin population for which data were available in Germany and France.

Political elites are increasingly unlikely to have a working-class background (e.g., Bovens and Wille 2017). For this group—just as for women and immigrants—the lack of time, networks, and money matter as central resources to (successfully) seek office (Carnes 2018). Class is a widely used notion in social science research. To capture political inequality, class is operationalized as occupational or educational attainment; sometimes a similar argument is made regarding income (Giger et al. 2012). Due to the strong differences in educational systems in France and Germany and the resulting problems of comparability, we focused on occupation as the indicator for class and measured the share of workers as wage earners.^3^

Finally, access to political decision-making positions is shaped by age restrictions. Typically, the right to stand in elections is given to individuals who have attained their 18th birthday. However, the middle-aged dominate legislatures. Relatively better representation of younger age groups in some countries has been causally linked to the surge of parties in which they are overrepresented (Stockemer and Sundström 2018; Freire et al. 2022). We thus take the share of those between 18 and 30 years old to assess the representation gap of younger citizens.

### Operationalization of the Criteria Used to Assess the Effectiveness of the Fora to Balance Representation Gaps

Above, we presented three minimum criteria for the institutional design of such fora in order to balance representation gaps. We operationalized the first criterion, “composition,” via the number of participants, the process of selection, and—analogous to the selection of groups in parliaments—the representativeness of the forum in terms of gender, age, occupational status/education,^4^ and ethnic minority. The second criterion, “deliberative quality,” was assessed by the duration and the process of the forum. Given the absence of strong theoretical priors on “ideal” duration, we operationalized duration from an empirical average score based on the duration of face-to-face, hybrid, or online meetings (3.2 days) as well as the time stretch from the first meeting to the last meeting (4.2 weeks) in the OECD Database on Representative Deliberative Processes and Institutions.^5^ The third criterion, “coupling to politics,” was operationalized as the mandate, the number of recommendations adopted, and their impact.

## Representation Gaps in France and Germany

**Fig. 1:**
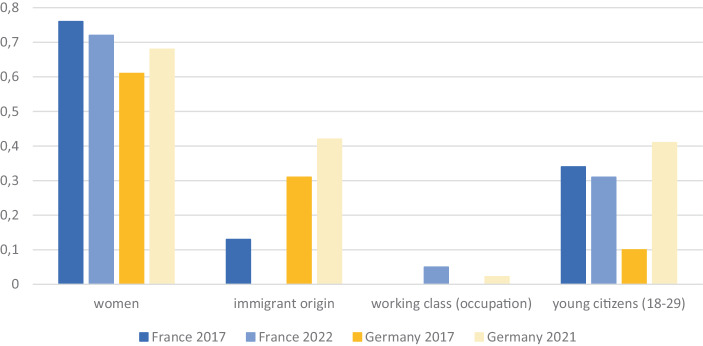
Representation indices (RIs) for 2017 and 2021/2022, France and Germany. A value of 1 indicates perfect representation, while a value less than or greater than 1 indicates under- or overrepresentation of the group, respectively (Bloemraad 2013). Note that the RI for working class is 0 in France in 2017 and in Germany in 2017. In addition, data for immigrant origin and working-class people in France in 2022 are missing. See Tables 2 and 3 in the Appendix

This section offers a comparative description of the various representation gaps that exist in the French and German parliaments. Figure 1 shows RIs for each of the selected groups. If not reported otherwise, we used public administration data to assess the proportion of a social group in the population as well as data on representatives in the French and German parliaments.^6^ We covered two points in time, 2017 and 2021/2022.

Figure 1 shows that none of the groups under study reached full descriptive representation (RI less than 1 for all groups). Differences between groups seem more pronounced than between the two countries.

In both France and Germany, the proportion of *women* living in the country was about 51%. In France, female representatives in the Assemblée Nationale accounted for 39% (2017) and 37% (2022) in the last elections, respectively. Germany is characterized by slightly lower proportions, 31% (2017) and 34.9% (2022). In both countries, the gender gap is persistent: In Germany, the RI was 0.61 in 2017 and 0.69 in 2021. In France, the RI was 0.76 in 2017 and 0.72 in 2022.

In 2015, 13.4 million people of *immigrant origin* lived in France (excluding Mayotte), which was 20.1% of the population. Yet the share of representatives was only 2.8% (Geese 2022, p. 7). This results in an RI of 0.14 for the 2017 election. No data were available for 2022. In Germany, according to the microcensus, the percentage of the immigrant-origin German population was 26% in 2019. The Mediendienst Integration provides information on politicians of immigrant origin. The data show that 83 of the 735 members of parliament (11.3%) of the 20th legislature in 2021 were of immigrant origin.^7^ Thus, while every fourth individual living in Germany was of immigrant origin, this was only true for every ninth MP. On this basis, we calculated an RI of 0.31 (2017) and of 0.42 (2021) for people with an immigrant origin.

Much like other European parliaments, the French Assemblée Nationale and the German Bundestag are parliaments of academics (Sineau and Tiberj 2007, pp. 175–176; Kintz and Cordes 2019)—*blue-collar workers* are hardly represented. In 2017, 5,585,000 blue-collar workers, or 20.7% of employees in France, were blue-collar workers. Yet not a single representative belonged to this social group in 2017. The newly elected Assemblée Nationale counts six working-class deputies. Consequently, we calculated RIs of 0 (2017) and 0.05 (2022). In Germany, 13.1% of the economically active population were blue-collar workers in 2020. The occupational structure in the German Bundestag is remarkably different: No blue-collar worker was represented in the 19th legislature, whereas two MPs of the 20th legislature were blue-collar workers (Kintz and Cordes 2019; Pyschny and Kintz 2022). Accordingly, the RI of the working class was 0 in 2017 and 0.02 in 2021.

Turning to *young citizens* (18–21 years), underrepresentation is strong, but differs between France and Germany. In the Assemblée Nationale elected in 2017, more than half of the seats (58%) were occupied by MPs between 40 and 59 years of age. Individuals aged 60 and older accounted for almost a quarter (23.6%). This is very different from the age structure of the French population, in which the groups aged 40–59 as well as those older than 60 made up 26% of the population, while those aged 18–29 made up 14% of the population. The age gap is particularly pronounced for the youngest. There were only 27 members younger than 30 (4.7% of members), resulting in an RI of 0.34 in 2017 and an even lower RI, 0.31, in 2022. The marked incumbency logic of the French electoral system might provide an explanation. The situation is different in Germany. In 2020, 54% of Germans were older than 50 years, and 16% were between 18 and 29 years old. By contrast, the share of the Bundestag members who were older than 50 years decreased to 46% in 2021, and 6.5% were younger than 30 (48 individuals). Compared with 2017, the proportion of young representatives increased remarkably from 1.6%. Yet it is still much lower than the proportion of Germans aged between 18 and 30, resulting in an RI of 0.1 in 2017 and 0.4 in 2021.

In comparing the two countries, the following findings are particularly noteworthy. First, in spite of great political awareness and several measures (e.g., legislative and party gender quotas), the gender gap still exists. Second, in both countries, the class gap is the most remarkable. We find that the trend might be changing in recent years, yet parliamentarians in France and Germany are still overwhelmingly academics and in much greater proportion than those they represent. Third, the ethnic minority gap is somewhat smaller in Germany than in France. This finding, however, must be interpreted with caution, as it is strongly influenced by the measure of immigrant origin we use. Finally, even though on average the German parliamentarians are younger than the population, the age representation gap seems to matter more in Germany than in France.

## Closing the Gaps—But How?

Government responses to representation gaps take place within existing political institutions; prominent examples are electoral reforms (Krook 2010; Rohrschneider and Thomassen 2020), as well as innovations outside established political institutions. These often take the form of deliberative participatory democracy (Bächtiger et al. 2018). The first experiments were done in the 1970s, developed over time, and became more widely visible with deliberative participatory fora on electoral reforms held in the Netherlands and Canada in the mid-2000s and on the constitutions in Ireland and Iceland in the early 2010s. The OECD (2021) Database of Representative Deliberative Processes and Institutions (1979–2021) displays 599 such initiatives, 19 of which have been initiated in France and 64 in Germany. We acknowledge that a strong causal argument would require more detailed analysis of governments’ motivation and other drivers for installing these deliberative participatory fora. Yet, we are confident that the claims made by government actors and secondary analysis are supportive to the causal connection we imply.

In France, deliberative participatory fora figure prominently in the debate about the quality of democracy. In the run-up to the 2017 presidential election, several candidates called for these democratic innovations (Benoît Hamon of the Socialist Party, Emmanuel Macron of *En Marche/Renaissance*, and Jean-Luc Mélenchon of *La France Insoumise*). They proposed different institutional designs such as an annual audit of the president or citizens drawn by lot in the senate. They pledged that resulting proposals should become policies. The proposal for “a great permanent debate” was revived in the run-up to the 2022 presidential election campaign, promising to render citizen participation and deliberation a permanent feature of the French political system (Gatinois 2022). Also in Germany, the government is considering instruments that aim at ensuring a more comprehensive inclusion and better representation of the people. The Coalition Treaty of 2021 between the Social Democratic Party, the Greens, and the Liberals states the coalition’s will to commission citizen councils to work on specific issues and to ensure equal participation and for the parliament to consider the results.

To allow for a comparative assessment, we will focus our analysis on cases at the national level. For France, we investigated the *Grand Débat National* (Great National Debate; GDN) and the *Convention Citoyenne pour le Climat *(Citizen Assembly for Climate; CCPC), the two most prominent deliberative participatory fora that have been widely covered in the grey literature and allow assessment of our question about whether these innovations allow closing of the representation gap. For Germany we investigated the *Bürgerrat Deutschlands Rolle in der Welt* (Germany’s Role in the World; DRW), arguably the most prominent national citizen assembly. In addition, we examined the *Bürgerrat Klima* (Citizen Council Climate; BK), which thematically matches the CCPC. Assuming that deliberative participatory fora have the potential to balance political inequality, we ask how they should be designed institutionally.

In the following, we give a short overview of the context in which the four deliberate–participatory fora were set up before we discuss institutional design features along with the three criteria.

### Grand Débat National^8^

The *Grand Débat National* is a direct response to the *Mouvement des Gilets Jaunes* (Yellow Vest Movement). The movement formed against economic and fiscal injustice and called for more democracy in the political system. It mobilized many citizens who had previously participated little in politics, had lower education, and were economically less well off than average French citizens (Grossman 2019, p. 33). Facing a numerically strong and in parts violent movement, the government did not only give in to some of the substantial demands but also opened an alternative channel for people to participate in politics, and it announced the GDN on December 10, 2018.

The GDN realized 19 regional and four thematic conferences on tax and fiscal policies, public administration and services, energy transition, and democracy and citizenship, which took place on different weekends in March 2019. For these deliberative participatory fora, a total of 1404 citizens were selected in a two-stage process. First, 300,000 people were randomly selected based on mobile phone numbers, and participants were then randomly selected from those who had expressed interest in participating. The resulting fora were far from representative. Based on participant characteristics from 18 regional conferences, we calculated RIs close to 1 for women (0.9), young citizens (0.6), and those in the working class (0.2).^9^ Including the 19th regional conference in the calculation of the RI, which exclusively focused on young citizens, would further reduce the representation gap for this group. The representation gap remains greatest for people from the working class, with participants having higher incomes and higher education being strongly overrepresented. Overall, the deliberative participatory forum moved toward closing the existing representation gaps but stayed far from balancing them.

The GDN was equipped with important institutional features impacting the deliberative quality. To animate and structure the debates, central organizers provided the participants and the local facilitators with background information and discussion points along the lines of the four themes. The participants and the facilitators had to respect norms of deliberation, such as respectful interaction and attention to neutrality toward opinions. A feature critically affecting the quality of deliberation was the rush in which the GDN took place. Each deliberative participatory forum took place during one weekend, leaving little room for deliberation to unfold.

The link to politics is characterized by a mandate given by the French president to discuss the four central themes listed previously. Given its strong visibility in the French media and its appearance as a response to the protests of the Yellow Vest Movement, it is not surprising that the 146 recommendations adopted by the CCPC were taken to the Assemblée Nationale and the Sénat. President Macron announced reforms flowing from the GDN in a speech on April 25, 2019. Yet, to date, few concrete reactions are observable. They mostly concern projects complementing existing government priorities, such as a law strengthening local government (*Engagement et Proximité*), territorial decentralization (*Loi “3DS”*) and rearrangements of services of general interests (*Maisons de services aux publics*), reform of elite education (*ENA*), and strengthening of participatory democracy, which resulted in the CCPC (see below). Thus, while some “cherry picking” (Font et al. 2018) took place, most of the recommendations have so far had no practical impact (Keller 2019, p. 13).

### Convention Citoyenne pour le Climat^10^

A second innovative forum is the *Convention Citoyenne pour le Climat*. The CCPC’s mandate is to define “structural measures to achieve, in a spirit of social justice, a reduction of greenhouse gas emissions by at least 40% by 2030 compared to 1990 levels.” The CCPC shows some similarities to the GDN: It was composed of 160 citizens drawn by lot. In a first stage, approximately 225,000 randomly selected citizens were contacted by phone, and from those who showed interest in participating, a representative panel was formed, taking into account gender, age, education, and socioprofessional and geographic situation. The number of participants varied slightly across the seven sessions, but without affecting our RIs (women, RI 1; young citizens aged 18–34, RI 0.9; and working class, RI 0.5) (Fourniau et al. 2020, pp. 7–8).^11^ Compared to the earlier experience with the GDN, the CCPC substantially improved mirroring of French society and closed representation gaps for women and young citizens.

Regarding support of the quality of deliberation, a central difference to the GDN is that the CCPC deliberations were accompanied by 52 scholars who presented insights from research and were available for questions. The CCPC met in seven sessions from October 2019 to June 2020 (including two virtual meetings due to the lockdown in France in spring 2020), thereby allowing the process to unfold over time. Deliberation took place in five thematic working groups (housing, work, production, transport, consumption) and in plenary sessions on cross-cutting issues such as constitutional consequences and financing aspects. The work concluded with a final vote by majority.

Turning to coupling to politics, we note that unlike the broad thematic outreach of the GDN, the CCPC held a mandate to define measures aiming at greenhouse gas reduction (40% by 2030) while paying attention to social justice. In a press conference on April 25, 2019, President Macron announced that the proposals emerging should be put to a referendum “without filter” or to a vote in parliament or be applied directly. A total of 149 recommendations were adopted, and a nongovernmental organization called *Les 150—L’association des Citoyens de la Convention Climat *was created to monitor follow-up. A broad reform bill has been announced to take up some of the 149 recommendations. This, however, got stuck in political struggles aggravated by the COVID-19 pandemic and the incipient election campaign, further weakening the (selective) legislative answer to the CCPC.

### Deutschlands Rolle in der Welt^12^

In 2019, the civil society association Mehr Demokratie and the Schöpflin Foundation organized the first so-called Citizens’ Council Democracy headed by Bavaria’s former prime minister, Günther Beckstein. This first Bürgerrat (citizens’ council) prompted another Bürgerrat in 2020 that dealt with DRW. The range of topics included sustainable development, economy and trade, peace and security, democracy and the constitutional state, and the EU.

The 160 participants were drawn by lot, but the three-stage selection process had to fulfill criteria of territorial and sociodemographic diversity: First, the organizers asked 84 municipalities from all German regions and of different sizes to provide address data drawn at random. From those municipalities that responded positively (68), 4365 individuals were invited to participate. The organizers then selected participants who were representative of the population in terms of age, education, size of place of residence, country of origin, and gender. While group representation worked well in the gender and age categories (i.e., RIs of 1 or higher), the group of low-educated persons was severely underrepresented: Instead of having about 30% of the participants with no degree or a lower secondary school degree, only 10% of the Bürgerrat had a lower secondary degree, and only 0.6% (instead of 4%) had no school degree (RI: 0.6). In addition, 13.6% of the participants had a migration background compared with 26% in the general population (RI: 0.5).

The institutional set-up supported the quality of deliberation in the following respects. The participants met virtually in 10 meetings in January and February 2021 to work on recommendations. They were supported by professional moderators and experts from diverse backgrounds, e.g., science, media, and society. The experts’ role was to inform the participants on discussion topics.

Coupling to politics was comparably strong because the DRW was initiated by the Bundestag. Together with social actors and research institutes, MPs from all parties drafted guiding questions. These questions were checked both in focus groups with randomly selected citizens and via a representative survey of the population. Finally, a workshop with political and societal representatives developed the final program for the Bürgerrat. Besides the Bundestag’s interest regarding the substance, the Bürgerrat was also seen as an experiment to test whether such an instrument would be suited for complementing classic legislative work.^13^ In March 2021, the president of the Bundestag received the final report. Until the federal election in September 2021, participants explained their recommendations in several workshops and talks at federal ministries or meetings in parliament. While parties expressed high interest and willingness to incorporate proposals into their manifestos, until now no concrete reactions to the results of the DRW have been observable.

### Bürgerrat Klima^14^

The *Bürgerrat Klima* took place after the DWR had finished. In contrast to its predecessor, it was not initiated by political actors in office but by civil society organizations. They used the DWR as a blueprint: A scientific advisory board worked out thematic parameters, parliamentarians and civil society associations were asked to prioritize and identify topics and questions, and a survey was conducted to assess people’s opinions on the importance of themes.

Regarding composition, the 160 participants were drawn by lot in a two-stage process: Individuals older than 16 were randomly selected based on phone numbers. Those who showed interest were invited by mail to apply for participation. The final round of participants was then chosen based on characteristics such as age, gender, migration background, educational attainment, and size of hometown. The BK featured RIs of 1 in three of the four categories. Only the working class was underrepresented: 18% of participants were reported to have the lowest educational qualification (*Hauptschulabschluss*) compared with 29.6% in the general population (RI: 0.6) (Autorengruppe Bildungsberichterstattung 2021).

Turning to features supporting quality deliberations, the participants met in 12 virtual sessions taking place between April and June 2021 to debate four fields of action related to the challenges of climate change and how Germany could reach the agreed temperature goal: mobility, food, buildings and heating, and energy. Each session was opened with inputs by researchers. Scholars were also available to check facts and to answer questions from the participants.

In addition, much importance was placed on a close relationship to politics before and after the meetings. The final report was delivered before the elections in September 2021. A survey showed that 80% of the population thought that political actors should take the recommendations into account. As with the DWR, however, concrete action is to date not observable, even though all parties showed great interest in the recommendations. Coalitional dynamics in government are likely to have a greater impact on the choice of policy proposals.

### Comparative Assessment

Table 1 summarizes core features of the citizen assemblies relevant to assess the potential to balance the identified representation gaps regarding composition, high-quality deliberation, and a link to politics.

**Table 1:** Deliberative participatory fora in Germany and France

Key aspects	Grand Débat National	Convention Citoyenne pour le Climat	Bürgerrat Deutschlands Rolle in der Welt	Bürgerrat Klima
**Descriptively representative composition**				
*Number and selection*	1404 in 21 assemblies of 19–125 participants (random selection of phone numbers and stratified sampling according to five criteria)	160 (sortition and stratified sampling selection according to six criteria)	169 (sortition and stratified sampling selection according to six criteria)	160 (sortition and stratified sampling selection according to six criteria)
*Representation index*	Women 0.9, young citizens 0.6, working class 0.2, immigrant origin not available	Women 1, young citizens 0.9, working class 0.5, immigrant origin not available	Women 1, young citizens 1, working class 0.6, immigrant origin 0.5	Women 1, young citizens 1, working class 0.6, immigrant origin 1
**Quality of deliberation**				
*Duration*	One meeting, 1.5 days in groups of five to seven in March 2021	Seven weekend meetings and one online meeting, stretching over 9 months (October 3, 2019, to June 21, 2020)	10 online meetings in groups of six to eight, stretching over 10 days (January 13 to February 20, 2021)	Nine meetings in small groups, three plenary meetings, stretching over 12 days (April 26 to June 23, 2021)
*Role of experts*	Input	Input	Could be consulted	Input
**Coupling to politics**				
*Number and impact of final recommendations*	Mandated by President 146 measures debated in parliament and by government, selective uptake in reforms	Mandated by President 149 measures, promise to turn them into law Monitoring of follow-up by nongovernmental organization	Mandated by parliament Five policy guidelines and 32 recommendations, promise to deal with them in plenary sessions and committees	Mandated by civil society organization 10 guiding principles, 83 recommendations

Regarding the participant selection, all four citizen assemblies used sortition and stratified sampling selection. The French GDN, however, reproduced some of the existing representation gaps, notably for younger citizens, ethnic minorities, and working-class individuals. This is particularly disappointing because its much bigger size would have allowed mirroring of different social groups more easily. In contrast, the CCPC and both German Bürgerräte worked with stratified selection that shows a greater potential to balance existing representation gaps. Unlike the Assemblée Nationale, working-class citizens were represented in the CCPC, and age groups mirrored French society. Both the German Bürgerrat DRW and the BK failed to represent the society’s share of workers, whereas the other groups we studied were well represented in the DRW. The proportion of people of immigrant origin was also lower in the BK than in the general German population.

The conditions for quality of deliberations differed regarding information, expert input, moderation, and time accorded. All fora provided information for participants, and exchange was organized by facilitators. Expert input on demand by researchers who accompanied the debates was ensured continuously for the CCPC, but research input was also provided in the DRW and BK.

Turning to duration, the GDN stands out with the shortest time span regarding the duration of meetings reserved for deliberation (1.5 days) as well as the time from the first to the last meeting, as there was only one meeting per group. The CCPC marks the other end of the continuum, with seven meetings each lasting a weekend and stretching over a period of 7 months. Yet the number of meetings was about the same. Overall, all fora had institutions in place that aimed at high quality of deliberation, but we need more detailed information on the implementation of these institutions to assess whether voices of all groups represented did find their way into aggregated outputs (Gherghina et al. 2021).

The features that couple the deliberative participatory fora to politics are more difficult to assess comparatively. First, on the input side, mandates differed substantially. Two fora, the BK and the CCPC, focused on climate. The GDN and the DRW were more encompassing, taking up a range of topics of societal and political relevance. While the French mandates were both given by the president, in Germany only the DRW held a clear mandate by the Bundestag. Second, in the German political system, the output in the form of recommendations was directed at the parliament—which in the case of the DRW was also important in setting up the forum in the first place. In the French political system, recommendations have been officially received by the president but were also debated in parliament. Yet take-up of recommendations is subject to picking and choosing (Font et al. 2018), which is already evident despite the fact that some of the citizen assemblies finished their work only recently, with input still being debated on ensuing legislative reforms.

In summary, in particular the composition of the four studied deliberative participatory fora clearly mirrors society better than the parliaments studied. The institutional features guaranteeing quality of deliberation partially show potential to balance the representation gap. But this potential is critically limited by the lack of an institutional mechanism for a link to politics.

## Discussion

Our article provides a conceptualization and detailed description of representation gaps for women, people of immigrant origin, the working class, and younger age groups in France and Germany. Comparing two countries and four groups allows us to see that important representation gaps exist for all groups, but the gap is most pronounced for class. In addition, the representation gap for gender and people of immigrant origin seems to be narrowing over time. Regarding age in France, deputies are, on average, substantially older than the population as well as their counterparts in Germany.

The comparison of the French and German cases further indicates that despite the fact that group-specific gaps are influenced by the interplay of electoral system features (e.g., women quotas) and cultural aspects (e.g., notions of citizenship), the countries show largely similar patterns across the different groups analyzed. This leads us to expect that at least among other Western European countries, the representation gap for women is likely to be smaller compared to the other groups studied, particularly social class. What is more, these differences in gaps and trends highlight that we should engage systematically with potential trade-offs in the representation of groups. Future research should concentrate on identifying the drivers of representation of individuals with intersecting categories of underrepresented groups. This requires research to engage in systematic data collection that will help fill these knowledge gaps.

Deliberative participatory fora hold some potential to tackle underrepresentation by opening new channels of participation. This potential is critically influenced by the institutional design of these fora. We explored this potential empirically in four recent cases of deliberative participatory fora: the *Grand Débat National*, formed as a deliberative answer to the Yellow Vest movement, and the *Convention Citoyenne pour le Climat* in France and the *Bürgerräte *in Germany to support policymakers in decision-making processes. Strikingly, country differences seem negligible. Rather, the comparison allows a clear assessment of what already works well and where more efforts are needed to benefit from the potential of balancing underrepresentation: Random stratified sampling assured largely descriptive representation in three of our four cases. Yet, despite efforts, working-class people are still underrepresented. This holds the potential danger of further discouraging them from politics, as their interests not only are absent in parliamentary debates but are not even heard in fora explicitly designed to give underrepresented groups a voice. All fora are designed such that institutions support the quality of the deliberative process by offering information and expertise to participants as well as providing moderation by professional facilitators. However, other institutional features (e.g., time for deliberation) could be improved to utilize more fully the potential for quality deliberations. Finally, while political support was important to the set-up of all four cases, our findings clearly show that this does not mean that governments want to share power with ordinary people. While in all cases a political mandate of more or less precision was given, in none of the cases did we find that institutions secured an uptake of the recommendations. We conclude that the biggest challenge for balancing representation gaps with deliberative participatory fora in the future is related to the institutional design of coupling—even if a somewhat improved take-up of recommendations might still occur in the future. Recent research shows that this can easily aggravate political dissatisfaction rather than curing current challenges to liberal democracy (van Dijk and Lefervre 2022). Differences between our cases further suggest that institutional approaches to address this challenge depend on the political system. Our cases in Germany show that in a parliamentary system, coupling could benefit from institutionalized links to parliament, e.g., via specific committees that include representatives from parliament and from citizen fora—something other parliamentary systems have experienced positively (Landemore 2020). The French case, in turn, highlights the important role of the president. Here, institutional features to address the challenge should incentivize (e.g., by creating co-ownership) or force (e.g., veto power of the citizen forum in cases of far-reaching changes to proposals) the president to follow the input of citizens. While we would not go so far as to argue that efforts are superfluous, we believe that a *stronger* government affirmation of an uptake of the recommendations would signal much more willingness to include underrepresented groups’ voices and not leave some groups disaffected.

The dominant challenge of coupling is likely to matter for a much greater number of cases as it links to the inherent tension between features of representative political institutions and raises more fundamental questions on the balancing of underrepresentation within parliament through extraparliamentary channels. Direct participation (of some) sits uneasily with the logic of representative democracy more generally. While deputies are accountable to their voters and can be sanctioned in the next elections, no such “control” mechanisms exist for citizen fora. Processes within deliberative participatory fora are frequently less transparent to ordinary citizens than are parliamentary debates and votes. Arguably, this lack of transparency and accountability in the decision-making chain negatively affects the legitimacy of a political system more generally. We might also generalize that more balanced representation within political institutions should be given priority to achieve political equality. This does not disregard the potential of participatory–deliberative fora to contribute to the quality of democracy and the functioning of politics in other respects. Lafont (2017) argues that rather than giving citizen assemblies decision power, they should be a tool to challenge the majority opinion of the public, to enhance the agenda-setting power of ordinary citizens or anticipate what the public would want if it were more informed and deliberative. Thus, future research should discuss the benefits and costs of improving descriptive representation externally—ideally by comparing different external channels such as direct democracy (Trüdinger and Bächtiger 2022) and deliberative participatory fora.

## Appendix

**Table 2:** Calculation of representation indices (RI) for parliament, France

	% population 2017 (a)	% population 2022 (b)	% deputies 2017 (c)	% deputies 2022 (d)	RI 2017 (=c/a)	RI 2022 (=b/d)
Women	51.6	51.6	39	37	0.76	0.72
People of immigrant origin	20.1	21.2	2.8	Not available	0.14	Not available
Working class (occupation)	19.2	20.7	0	1	0	0.05
Young citizens (18–29)	14	14	4.7	4.3	0.34	0.31

Sources: See sources provided in the text

**Table 3:** Calculation of representation indices (RI) for parliament, Germany

	% population 2017 (a)	% population 2022 (b)	% deputies 2017 (c)	% deputies 2022 (d)	RI 2017 (=c/a)	RI 2022 (=b/d)
Women	51	51	31	35	0.61	0.68
People of immigrant origin	26	26.7	8	11.3	0.31	0.42
Working class (occupation)	21.4	13.1	0	0.3	0	0.022
Young citizens (18–29)	16	16	1.6	6.5	0.1	0.41

Sources: See sources provided in the text

## Footnotes

1 Elsässer and Schäfer (2022) ground their argument in the theoretical distinction of symbolic and substantive representation. The working class might not suffer from a historical image to be “ill-fit to participate” or “unable to rule” that could be remedied by better symbolic representation, but rather from an empirically more recent lack of responsiveness to their substantial interests.

2 We leave out criteria regarding purpose and features of the wider setting, such as website transparency or conversation with the wider public. These things are normatively important but have little potential to directly address unbalanced participation. Also, depending on how detailed the lists are, several criteria inform one theoretical argument.

3 We are aware that today many workers in the service sector as well as self-employed individuals share characteristics of the working class, but we had to exclude them due to lack of available comparative data.

4 Information on occupational status is not always provided for the deliberative participatory fora, but participants’ educational attainment is reported.

5 https://www.oecd.org/governance/innovative-citizen-participation (last accessed December 14, 2022).

6 For France, data are collected from the homepages of the national statistical office, INSEE (https://www.insee.fr/fr/statistiques; last accessed August 30, 2022), and Assemblée Nationale (https://www.assemblee-nationale.fr/dyn/vos-deputes; last accessed January 18, 2023). For Germany, we consulted the federal statistical office, Statistisches Bundesamt, notably the microcensus (https://www.destatis.de/DE/Themen/Gesellschaft-Umwelt/Bevoelkerung/Bevoelkerungsstand/_inhalt.html; last accessed September 5, 2022), and data for the Bundestag (https://www.bundestag.de/dokumente/parlamentsarchiv/datenhandbuch; last accessed September 5, 2022).

7 Mediendienst Integration (2021). Mehr Abgeordnete mit Migrationshintergrund. https://mediendienst-integration.de/artikel/mehr-abgeordnete-mit-migrationshintergrund-1.html (last accessed September 5, 2022).

8 Unless noted otherwise, information was drawn from the official site, https://granddebat.fr/pages/ressources (last accessed December 14, 2022).

9 Calculated from data for 18 regional conferences, including data for young citizens aged 18–34 years. For people of immigrant origin, no numbers were available.

10 Unless noted otherwise, information was drawn from the official site, https://www.conventioncitoyennepourleclimat.fr/ (last accessed January 18, 2023).

11 No data were available for citizens of immigrant origin.

12 Unless noted otherwise, information was drawn from the official site, https://deutschlands-rolle.buergerrat.de (last accessed March 9, 2022).

13 Deutscher Bundestag (2020). Bundestag beschließt neue Form der Bürgerbeteiligung: Bürgerrat soll Parlament Bürgergutachten zur Rolle Deutschlands in der Welt vorlegen. Press release of June 18, 2020. https://www.bundestag.de/presse/pressemitteilungen/701614-701614 (last accessed 18.01.2023).

14 Unless noted otherwise, information was drawn from the official site, https://buergerrat-klima.de (last accessed September 5, 2022).
